# Sex Differences in the Progression of Metabolic Risk Factors in Diabetes Development

**DOI:** 10.1001/jamanetworkopen.2022.22070

**Published:** 2022-07-14

**Authors:** Yilin Yoshida, Zhipeng Chen, Robin L. Baudier, Marie Krousel-Wood, Amanda H. Anderson, Vivian A. Fonseca, Franck Mauvais-Jarvis

**Affiliations:** 1 Section of Endocrinology and Metabolism, Deming Department of Medicine, Tulane University School of Medicine, New Orleans, Louisiana; 2 Tulane Center of Excellence in Sex-Based Biology and Medicine, Tulane University, New Orleans, Louisiana; 3 Southeast Louisiana VA Medical Center, New Orleans, Louisiana; 4 Department of Biostatistics and Data Science, Tulane University School of Public Health and Tropical Medicine, New Orleans, Louisiana; 5 Department of Epidemiology, Tulane University School of Public Health and Tropical Medicine, New Orleans, Louisiana; 6 Section of General Internal Medicine, Deming Department of Medicine, Tulane University School of Medicine, New Orleans, Louisiana

## Abstract

This cohort study uses data from the Atherosclerosis Risk in Communities (ARIC) Study to investigate whether the increase in metabolic syndrome severity is more pronounced in women than men and whether the sex difference varies by race.

## Introduction

The mechanism underlying women's higher risk of cardiovascular disease (CVD) associated with diabetes is not fully elucidated but may be related to greater metabolic risk deterioration before diabetes in women than men.^1^ The sex disparity in diabetic CVD varies by race. Non-Hispanic Black (Black) women with diabetes have a higher prevalence of CHD and stroke than Black men, while in non-Hispanic White (White) individuals, there is a male predominance in diabetic CVD.^2^ In this study, we investigated whether the increase in metabolic syndrome severity, a continuous biomarker of the severity of metabolic derangement, during prediabetes is more pronounced in women than in men and whether the sex difference varies by race.

## Methods

In this cohort study, we used data from the Atherosclerosis Risk in Communities (ARIC) Study visits 1 to 4 from 1987 to 1998. ARIC is a prospective study of middle-aged adults in the US.^3^ We reported findings according to the Strengthening the Reporting of Observational Studies in Epidemiology (STROBE) reporting guideline. This analysis involved secondary, deidentified data that did not require review from the institutional review board at Tulane University.

Our primary outcome was the metabolic syndrome severity score derived from waist circumference, triglycerides, systolic blood pressure (SBP), high-density lipoprotein (HDL), and fasting glucose (FG), accounting for the weight of the contribution of each component by sex and race.^4^ Race was self-reported, and the categories included non-Hispanic Black and non-Hispanic White. Ethnicity was not considered in this study because only a small portion of ARIC participants self-reported as Hispanic, which did not provide sufficient power for statistical testing. Race was considered in this study because sex differences in diabetes progression has rarely been reported in Black and White individuals separately, and the sex disparity in diabetic cardiovascular disease varies by race.

The score was calculated for participants at visits 1 to 4 with a 3-year interval between visits. We performed linear mixed regressions to model the metabolic syndromescore and components over time among men and women across FG levels (normoglycemia, <100 mg/dL; prediabetes, 100-125 mg/dL; diabetes, ≥126 mg/dL, or with a diabetes diagnosis; to convert glucose to mmol/L, multiply by 0.0555), including a random intercept and a random slope to account for the within-subject correlation. We adjusted for age, education, smoking, and antihypertensive, lipid-lowering, and antidiabetes medications. We also adjusted for body mass index (calculated as weight in kilograms divided by height in meters squared) for outcomes of SBP, HDL, triglycerides, and FG. The SAS statistical software, version 9.4 (SAS Institute) was used for statistical analysis. Statistical significance was set at *P* < .05.

## Results

This study included 13 412 individuals, with 7414 women (55%) and 9983 White individuals (75%) (Table). During a median follow-up of 9 years, women had a significantly greater increase in mean (SD) metabolic syndrome severity vs men (0.098 [0.004] vs 0.074 [0.004]) in the prediabetes state, while men experienced a greater increase in the diabetes state (0.055 [0.005] vs women 0.04 [0.005]) (Figure, Table). Metabolic syndrome severity increased at a greater rate among White women vs White men in the prediabetes state (0.12 [0.004] vs 0.054 [0.004]) and diabetes state (0.06 [0.006] vs 0.043 [0.006]); however, in Black individuals, there were no significant changes in metabolic syndrome severity across FG levels (Figure, Table). Women had a greater increase in waist circumference vs men in prediabetes and diabetes states for White individuals and the prediabetes state for Black individuals (Figure, Table). Compared with Black men, Black women had a greater increase in triglycerides across FG levels (Figure, Table) and a greater increase in SBP in the prediabetes state (Figure, Table). Men had a greater adverse change in HDL vs women across FG levels in white individuals (Figure, Table). No significant sex difference was observed in the change of FG in the prediabetes state. During diabetes, men showed a greater increase in FG (Figure, Table).

**Table.** Characteristics and Changes of Metabolic Risk Factors by Sex and Race

Characteristics and changes	All (n = 13 412)			White (n = 9983)			Black (n = 3429)		
	Men (n = 5998)	Women (n = 7414)	*P* value^a^	Men (n = 4643)	Women (n = 5340)	*P* value^a^	Men (n = 1355)	Women (n = 2074)	*P* value^a^
**Characteristics**									
Age, mean (SD), y	54.4 (5.8)	53.7 (5.7)	<.001	54.5 (5.7)	53.9 (5.7)	<.001	53.8 (6)	53.2 (5.8)	.007
High school education or less, No. (%)	2988 (49.9)	4404 (59.5)	<.001	2134 (46)	3121 (58.5)	<.001	854 (63.2)	1283 (62.0)	.49
Current smoking, No. (%)	1662 (27.7)	1823 (24.6)	<.001	1143 (24.6)	1325 (24.8)	.80	519 (38.3)	498 (24.0)	<.001
Antihypertensive medications, No. (%)	1206 (20.1)	1734 (23.4)	<.001	783 (16.7)	895 (16.8)	.90	423 (31.2)	839 (40.5)	<.001
Lipid lowering medications, No. (%)	1102 (18.6)	1755 (23.8)	<.001	803 (17.4)	1127 (21.2)	<.001	299 (22.6)	628 (30.7)	<.001
Antidiabetic medications, No. (%)	264 (5.6)	377 (5.7)	.90	136 (3.6)	145 (3.0)	.10	128 (14.3)	232 (13.3)	.50
Metabolic syndrome z-score, mean (SD)	0.4 (0.9)	0.3 (1.3)	<.001	0.36 (0.8)	0.1 (1.1)	<.001	0.34 (1.4)	0.7 (1.7)	<.001
BMI, mean (SD)	27.4 (4.2)	27.6 (6)	.009	27.3 (3.9)	26.4 (5.4)	<.001	27.6 (4.9)	30.7 (6.5)	<.001
Waist circumference, mean (SD), cm	98.8 (10.9)	94.7 (15.4)	<.001	99.4 (10.3)	92.6 (14.5)	<.001	96.8 (12.9)	100.2 (16.3)	<.001
Triglycerides, mean (SD), mg/dL	133.2 (71.1)	118.5 (63.5)	<.001	138.9 (71.8)	123.1 (67)	<.001	113.8 (64.9)	106.7 (51.8)	<.001
SBP, mean (SD), mmHg	122.3(17.7)	119.9 (19.4)	<.001	120 (15.9)	116.8 (17.7)	<.001	130.2 (21.3)	128.2 (21.1)	.005
HDL cholesterol, mean (SD), mg/dL	45.1 (13.9)	58.0 (16.9)	<.001	43.3 (12.1)	57.9 (16.9)	<.001	51.4 (17.3)	58.1 (17.1)	<.001
Fasting glucose, mean (SD), mg/dL	108.7 (35.1)	106.8 (40.6)	.003	106.6 (27.9)	102 (28.5)	<.001	116.1 (52.3)	119 (60)	.10
**Annual change of metabolic risk factors across visits (SD)^b^**									
Metabolic syndrome z-score									
Normoglycemia	0.07 (0.02)	0.09 (0.02)	.001	0.04 (0.02)	0.1 (0.02)	.80	0.07 (0.05)	0.08 (0.04)	.40
Prediabetes	0.07 (0.004)	0.1 (0.004)	<.001	0.05 (0.004)	0.1 (0.004)	<.001	0.07 (0.01)	0.09 (0.01)	.10
Diabetes	0.06 (0.01)	0.04 (0.01)	<.001	0.04 (0.01)	0.06 (0.01)	<.001	0.09 (0.01)	0.03 (0.01)	.20
Waist circumference									
Normoglycemia	0.6 (0.2)	1.4 (0.3)	.46	0.7 (0.2)	1.7 (0.4)	.10	0.3 (0.5)	1.6 (0.6)	.91
Prediabetes	0.7 (0.05)	1.8 (0.06)	<.001	0.8 (0.05)	2.1 (0.07)	<.001	0.5 (0.1)	1.8 (0.1)	.01
Diabetes	0.7 (0.07)	1.5 (0.09)	<.001	0.7 (0.08)	1.8 (0.1)	<.001	0.6 (0.1)	1.7 (0.2)	.07
Triglycerides^c^									
Normoglycemia	0.03 (0.01)	0.09 (0.01)	<.001	0.04 (0.001)	0.1 (0.01)	<.001	0.02(0.03)	0.07 (0.02)	<.001
Prediabetes	0.04 (0.002)	0.09 (0.002)	<.001	0.05 (0.002)	0.1 (0.002)	.10	0.02 (0.01)	0.07 (0.004)	.07
Diabetes	0.03 (0.003)	0.08 (0.003)	.03	0.04 (0.004)	0.1 (0.004)	<.001	0.03 (0.01)	0.07 (0.004)	<.001
SBP									
Normoglycemia	1 (0.47)	0.9 (0.5)	<.001	1.1 (0.5)	0.6 (0.5)	<.001	2.2 (1.2)	2.51 (0.9)	.06
Prediabetes	1 (0.08)	0.8 (0.08)	.90	1.1 (0.08)	0.4 (0.08)	.10	2 (0.2)	2.44 (0.2)	.80
Diabetes	0.8 (0.1)	0.7 (0.1)	.20	0.8 (0.1)	0.3 (0.1)	.004	2 (0.3)	2.39 (0.2)	.20
HDL cholesterol									
Normoglycemia	−0.7 (0.4)	0.5 (0.4)	<.001	−0.3 (0.4)	0.6 (0.5)	<.001	−0.9 (0.9)	−0.01 (0.7)	.04
Prediabetes	−0.7 (0.06)	0.4 (0.07)	<.001	−0.4 (0.06)	0.4 (0.08)	<.001	−1 (0.2)	−0.02 (0.2)	.07
Diabetes	−0.9 (0.09)	0.5 (0.1)	<.001	−0.4 (0.1)	0.5 (0.1)	<.001	−1.3 (0.2)	0.27 (0.2)	.40
Fasting glucose									
Normoglycemia	2.2 (0.6)	0.8 (0.5)	.005	1.1 (0.5)	0.6 (0.4)	.08	3.3 (2.2)	0.3 (1.7)	.07
Prediabetes	2.3 (0.1)	0.8 (0.1)	.90	1.2 (0.1)	0.7 (0.09)	.40	3.3 (0.5)	0.3 (0.4)	.80
Diabetes	2.4 (0.2)	0.4 (0.2)	.02	1.5 (0.2)	1 (0.2)	.30	3.2 (0.8)	−0.9 (0.6)	.10

Abbreviations: BMI, body mass index (calculated as weight in kilograms divided by height in meters squared); HDL, high-density lipoprotein; SBP, systolic blood pressure.

SI conversion factor: To convert triglycerides to mmol/L, multiply by 0.0113; HDL cholesterol to mmol/L, multiply by 0.0259; glucose to mmol/L, multiply by 0.0555.

^a^ *P* for sex differences.

^b^ Atherosclerosis Risk in Communities Study visits 1 to 4 (1987 to 1998).

^c^ Log-transformed.

**Figure.**
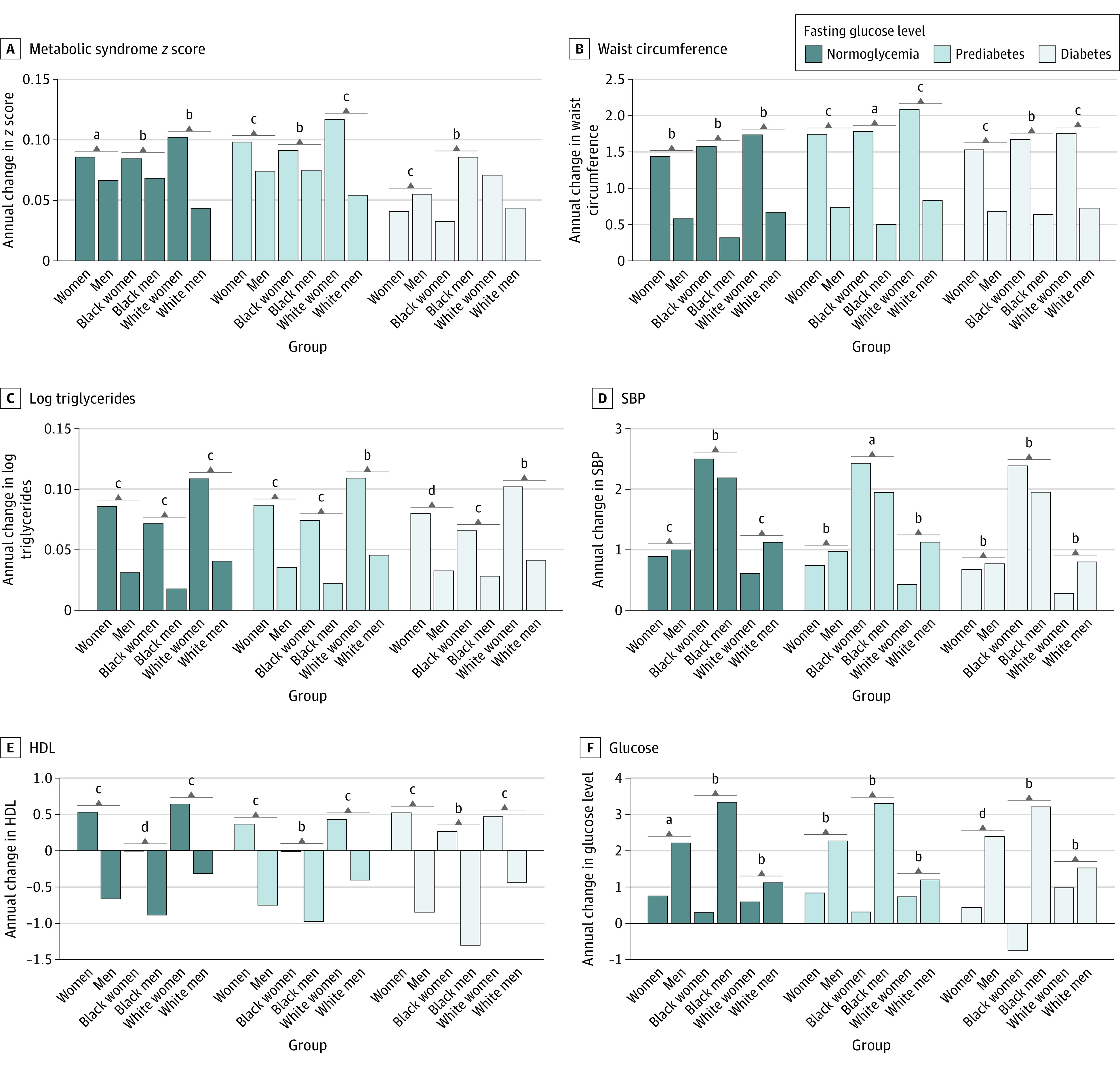
Annual Change of Metabolic Risk Factors Across Atherosclerosis Risk in Communities Visits HDL indicates high-density lipoprotein; SBP, systolic blood pressure. ^a^*P* < .01. ^b^Not significant. ^c^*P* < .001. ^d^*P* < .05.

## Discussion

In this study, women were associated with experiencing a greater increase in metabolic syndrome severity, central adiposity, and triglycerides than men in the prediabetes state, potentially explaining women's disadvantage in diabetic CVD risk. We extended prior cross-sectional evidence^5,6^ and highlighted women's worse metabolic risk profiles vs men before diabetes, and the sex difference varied by race. However, this study was limited because of the study’s short follow-up (9 years), so we cannot capture all individuals' 3-stage diabetes development (normoglycemia to prediabetes to diabetes). Sex and race-specific strategies for metabolic risk monitoring and control during prediabetes should be considered.
